# Factors associated with the use of psychotropic drugs by students at a brazilian public university

**DOI:** 10.17843/rpmesp.2024.414.13858

**Published:** 2024-11-26

**Authors:** Telma Regina Fares Gianjacomo, Camilo Molino Guidoni, Renne Rodrigues, Selma Maffei de Andrade, Jéssica Vertuan Rufino, Edmarlon Girotto

**Affiliations:** 1 Graduate Program in Public Health, State University of Londrina (UEL), Londrina-PR, Brazil. State University of Londrina Graduate Program in Public Health State University of Londrina (UEL) Londrina-PR Brazil; 2 Department of Pharmaceutical Sciences, State University of Londrina (UEL), Londrina-PR, Brazil. State University of Londrina Department of Pharmaceutical Sciences State University of Londrina (UEL) Londrina-PR Brazil; 3 Federal University of the Southern Border (UFFS), Chapecó Campus, Chapecó-SC, Brazil. Universidade Federal da Fronteira Sul Federal University of the Southern Border (UFFS) Chapecó Campus Chapecó-SC Brazil

**Keywords:** Medications, Psychotropic Drugs, Student Health, University, Brazil

## Abstract

**Objective.:**

To analyze the consumption of psychotropic drugs and their associated factors in university students, stratified by sex.

**Materials and methods.:**

We conducted a cross-sectional study with undergraduate students of a public university in Brazil. The instrument was an online self-administered questionnaire. The outcome variable was the use of psychotropic drugs, based on the Anatomical Therapeutic Chemical (ATC) classification system, and the exposure variables were socio-demographic, academic and health-related. The association between the exposure variables and the outcome was verified by calculating prevalence ratios and their 95% confidence intervals.

**Results.:**

We found that 12.2% (n=396) of the 3238 participants used psychotropic drugs; most were women (78.3%). The prevalence of psychotropic drug use was higher among students with access to health insurance, diagnosis of depression and diagnosis of anxiety. It was also higher in the group of women who reported using illicit drugs in the last three months and in the group of men who reported being dissatisfied or very dissatisfied with their academic performance. We detected low frequency of psychotropic drug use among women who consume alcohol on a weekly basis.

**Conclusions.:**

Our results show a higher frequency of psychotropic medication use in females, as well as in students with access to health insurance, diagnosis of depression and diagnosis of anxiety, regardless of sex.

## INTRODUCTION

Entering higher education is a transition that can cause stress, emotional imbalance, adaptation difficulties and a great impact on personal and academic life ^1^. Factors inherent to the academic process are pointed out as possible causes of health problems. During college, some students become vulnerable to the appearance of affective disorders, such as depression and anxiety ^2^. A systematic review with meta-analysis found that stress, low frequency of recreational activities, dissatisfaction with academic performance, and lack of emotional support in the academic environment are risk factors for mental health problems in undergraduate university students ^3^.

Recent research on the mental health of Chilean university students identified a significant proportion of students with mental health problems, estimating that 20-30% of this group is affected by anxiety, depression and stress. In addition, a notable gender disparity was reported, with a higher prevalence of depressive symptoms in women with 23.2%, compared to 13.4% in men ^4^. In this context, the mental health of college students has been a topic of interest in the literature ^5^. Studies reveal a high combined prevalence of depression and anxiety, among other mental health problems, in this population ^6^. Consequently, the use of psychotropic drugs among university students has increased, especially antidepressants, anxiolytics and psychostimulants, being also more frequent in women ^7^^-^^9^.

A study conducted among university students found a high prevalence of mental health disorders, with 30.4% of participants reporting the use of psychotropic medications, mainly for the treatment of anxiety (30%) and depression (22.8%). The study revealed that the use of psychotropic medications increases as students progress through their academic programs, particularly in the later years when academic pressure intensifies. In contrast, regular participation in physical activities and adherence to adequate sleep patterns were identified as protective factors against mental health disorders, underscoring the need for greater attention to the mental well-being of this demographic ^10^.

To better understand psychotropic medication use among students, it is necessary to consider the pressure of the academic environment, which contributes to stress and mental health impairment. In addition, it is essential to understand how individual factors, such as family background and resilience, interact with these stressors. It is also pertinent to analyze the relationship between psychotropic use and self-care behavior. A multidimensional approach that considers social and academic factors may help to develop more effective interventions to promote mental health among students ^11^^,^^12^.

However, solid research on this topic is scarce. Therefore, studies are needed to characterize the use of psychotropic drugs among university students and to correlate variables that help identify the groups most vulnerable to these drugs. In addition, some works have shown that the use of psychoactive drugs is higher in women, so it is important to evaluate the difference in the associated factors between male and female students ^13^^,^^14^. Therefore, this study aims to evaluate the use of psychotropic drugs in students of a public university in Paraná, Brazil, and to identify associated sociodemographic, academic and health factors, stratified by sex.

**KEY MESSAGES**

Motivation for the study. College students are exposed to numerous stressful events, which predispose them to problems such as depression and anxiety, leading to increased consumption of psychotropic medications.

Main findings. The use of psychotropic medications was reported by 12.0% of students, being higher among those with access to health insurance and diagnosed with depression and anxiety, as well as among those who reported using illicit drugs and who were dissatisfied with their academic performance. We found lower consumption of psychotropic drugs among women who consumed alcoholic beverages.

Implications. The evidence from this study may support actions to promote not only rational drug use campaigns, but also measures to minimize and help students with the stress of academic life.

## MATERIALS AND METHODS

### Study design, location, and population

This is a cross-sectional study, guided by the Strengthening the Reporting of Observational Studies in Epidemiology (STROBE) guidelines ^15^, analyzing data from the project “GraduaUEL - Analysis of Health and Life Habits of Undergraduate Students at UEL”. The overall objective of GraduaUEL was to analyze aspects related to health, exposure to violence and life habits of undergraduate students. The study population is composed of students at the State University of Londrina (UEL), in Paraná, Brazil, over 18 years of age, regularly enrolled in the first semester of 2019 in one of the 51 undergraduate courses. At the time of the survey, 12,536 students were eligible to participate ^16^.

### Pre-test and pilot study

The questionnaire prepared for this research was made available on the Google Forms® platform for students to complete. The questionnaire was divided into several thematic sections, covering general and academic characterization, life habits and sleep quality, medication use, experiences of violence, social support and resilience, mental health, and body satisfaction. To ensure its validity for application within the study population, the instrument was evaluated by experts in epidemiological research. A pre-test was conducted with 25 undergraduate students in the health area at a private institution in the city of Londrina, Brazil, to evaluate the clarity of the questions. In addition, a pilot study was conducted with 25 students from a federal institute of higher education in the region of Londrina, Paraná. This stage was conducted to verify the logistical conditions for data collection, such as response time and platform performance during simultaneous access.

### Data collection

Data were collected between April and June 2019. During this period, the researchers promoted the study at UEL, in all undergraduate classes, providing the link to access the questionnaire. The research was also a widely promoted in social networks, local press and mass emails sent to the university community. The questionnaire was answered anonymously, and the option to complete the enrollment number was optional for future individual feedback. Participants were instructed to answer the questionnaire only once. In addition, before students consented to participate in the study, they were asked, on the initial page of the electronic questionnaire, whether they were enrolled in an undergraduate program. If the answer was “no,” the questionnaire options were not displayed. In cases where duplicate or triplicate responses were identified, only the first response was considered. When the enrollment number indicated that the students were graduate students, they were excluded from the study.

### Outcome variable

The use of psychotropic medications was the outcome variable, and it was assessed by the following question: “Do you take any medication for continuous use?” If the answer was affirmative, the name of the medication, the person responsible for the prescription, and the period of use were requested. After data collection, the drug names were standardized to their generic name and categorized according to the World Health Organization’s Anatomical Therapeutic Chemical (ATC) classification system, considering the following subgroups as psychotropics: N05A (antipsychotics), N05B (anxiolytics), N05C (hypnotics and sedatives), N06A (antidepressants), N06B (psychostimulants and ADHD agents and nootropics) and N06D (antidementia drugs) ^17^. The drugs used were double-checked by different researchers and then analyzed with Epi Info software, version 3.5.1. In case of discrepancies, a third researcher made the necessary corrections.

### Exposure variables

Sociodemographic, academic, lifestyle and health-related variables were used as exposure variables. The assessed sociodemographic variables were: age (years), marital status (with partner-married or in stable union; without partner-single, divorced or widowed), self-reported skin color (white; non-white-yellow, multiracial, black or indigenous) and access to health insurance (yes; no). The analyzed academic variables were: study shift (morning/afternoon; evening; night; full-time/LDE-Long-distance education), year of study (1st year; 2nd or 3rd year; 4th, 5th or 6th year) and satisfaction with the course, and academic performance (very satisfied/satisfied; neither satisfied nor dissatisfied; dissatisfied/very dissatisfied). Finally, the variables related to lifestyle and health habits were: self-reported physical and mental health status (very good/good; fair; poor/very poor), self-reported sleep quality (very good/good; poor/very poor), self-reported medical diagnosis of depression and anxiety (yes; no), alcohol consumption (never/once or twice/occasionally; weekly; daily or almost daily) and use of illicit substances in the last three months (yes; no or prefer not to answer). The selection of variables to be assessed as associated with psychotropic medication use was based on the literature, especially studies related to mental disorders ^6^^,^^18^, which serve as indicators for the use of psychotropic substances. The exception was academic variables, which were selected based on variables available in the research.

### Statistical analysis

The data were analyzed using the Statistical Package for the Social Sciences (SPSS® version 19.0). In order to characterize the study population, a descriptive analysis was performed, presenting the frequencies of quantitative variables categorized by sex (male and female) ^19^. Mean age and standard deviation were also calculated for each sex independently. Poisson regression with robust variance was used to analyze the association between outcome and exposure variables, and to obtain prevalence ratios (PR) as a measure of association and 95% confidence intervals (95%CI). Crude (or bivariate) and adjusted analyses were performed, including all independent variables. All independent variables were included in the adjusted model, considering that, except for the academic variables, all are supported by the literature as being associated with mental disorders or psychotropic medication use. The significance level was 5% (p-value<0.05).

### Ethical considerations

All research subjects agreed to participate in the study, as it was only possible to answer the questionnaire if they accepted the informed consent form. This study was approved by the Human Research Ethics Committee (CEP) of the UEL (CAAE no. 04456818.0.0000.5231).

## RESULTS

**Figure 1:**
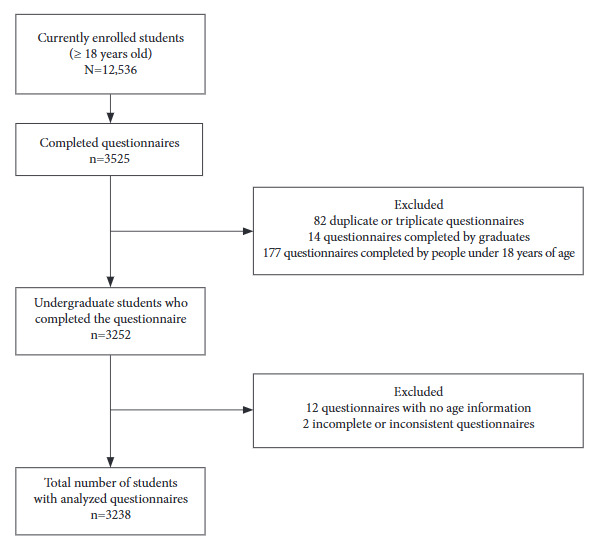
Flow chart of the GraduaUEL study sample, 2019.

A total of 3238 students were included according to the selection procedure shown in Figure 1. The majority of students participating in the study were self-reported white and unmarried. Males showed higher percentages of self-reported good physical health status, sleep quality, and mental health compared to females (Table 1).

**Table 1:** Description of academic variables, life habits and health conditions of university students, according to sex. GraduaUEL, 2019.

Independent variables ^a^		Women		Men	
		Total n=2221	Use of psychotropic drugs n=310 (78.3%)	Total n=1017	Use of psychotropic drugs n=86 (21.7%)
		n (%)	n (%)	n (%)	n (%)
Age (mean ± standard deviation)		21.76 ± 4.37	22.55 ± 4.90	22.28 ± 4.67	22.17 ± 3.74
Marital status (n=3238)					
	With partner	1107 (49.8)	158 (14.2)	413 (40.6)	51 (8.4)
	Without partner	1114 (50.2)	152 (13.7)	604 (59.4)	35 (8.5)
Self-reported skin color (n=3236)					
	White	1565 (70.5)	228 (14.6)	693 (68.1)	59 (8.5)
	Non-white	654 (29.5)	82 (12.5)	324 (31.9)	27 (8.3)
Access to health insurance (n=3235)					
	No	1194 (53.8)	138 (11.6)	591 (58.2)	41 (6.9)
	Yes	1025 (46.2)	171 (16.7)	425 (41.8)	45 (10.6)
Study time (n=3236)					
	Morning/afternoon	642 (28.9)	82 (12.8)	235 (23.1)	17 (7.2)
	Night	582 (26.2)	83 (14.3)	337 (33.1)	21 (6.2)
	Full-time/LDE (long-distance education)	995 (44.9)	145 (14.6)	445 (43.8)	48 (10.8)
Year of study (n=3226)					
	1st year	477 (21.5)	56 (11.7)	243 (24.0)	14 (5.8)
	2nd or 3rd year	1004 (45.4)	133 (12.2)	473 (46.8)	46 (9.7)
	4th, 5th or 6th year	733 (33.1)	129 (16.4)	296 (29.2)	26 (8.8)
Satisfaction with the course (n=3235)					
	Very satisfied/satisfied	1598 (72.0)	222 (13.9)	760 (74.8)	55 (7.2)
	Neither satisfied nor dissatisfied	496 (22.4)	60 (12.1)	175 (17.2)	18 (10.3)
	Dissatisfied/very dissatisfied	125 (5.6)	28 (22.4)	81 (8.0)	13 (16.0)
Satisfaction with academic performance (n=3236)					
	Very satisfied/satisfied	916 (41.2)	104 (11.4)	456 (44.9)	25 (5.5)
	Neither satisfied nor dissatisfied	756 (34.1)	106 (14.0)	324 (31.9)	33 (10.2)
	Dissatisfied/very dissatisfied	548 (24.7)	110 (18.2)	236 (23.2)	28 (11.9)
Self-reported physical health status (n=3238)					
	Very good/good	785 (35.3)	78 (9.9)	486 (47.8)	31 (6.4)
	Regular	952 (42.9)	134 (14.1)	379 (37.3)	30 (7.9)
	Bad/very bad	484 (21.8)	98 (20.2)	152 (14.9)	25 (16.4)
Self-reported mental health status (n=3238)					
	Very good/good	715 (32.2)	47 (6.6)	475 (46.7)	20 (4.2)
	Regular	848 (38.2)	101 (11.9)	325 (32.0)	26 (8.0)
	Bad/very bad	658 (29.6)	162 (24.9)	217 (21.3)	40 (18.4)
Self-reported sleep quality (n=3238)					
	Very good/good	1171 (52.7)	131 (11.2)	581 (57.1)	42 (7.2)
	Bad/very bad	1050 (47.3)	179 (17.0)	436 (42.9)	44 (10.1)
Medical diagnosis of depression (n=3236)					
	No	1919 (86.5)	123 (6.4)	935 (91.9)	41 (4.4)
	Yes	300 (13.5)	187 (62.3)	82 (8.1)	45 (54.9)
Medical diagnosis of anxiety (n=3236)					
	No	1444 (65.0)	50 (3.5)	799 (78.6)	19 (2.4)
	Yes	777 (35.0)	260 (33.5)	218 (21.4)	67 (30.7)
Alcohol consumption in the last three months (n=3238)					
	Never/once or twice/monthly	1527 (68.8)	219 (14.3)	619 (60.9)	58 (9.4)
	Weekly	647 (29.1)	81 (12.5)	362 (35.6)	21 (5.8)
	Daily or almost daily	47 (2.1)	10 (21.3)	36 (3.5)	7 (19.4)
Illicit drug use in the last three months (n=3238)					
	No/prefer not to answer	1698 (76.5)	217 (12.8)	695 (68.3)	56 (8.1)
	Yes	523 (23.5)	93 (17.8)	322 (31.7)	30 (9.3)

a The total number of respondents for some variables was different from the total number of participants (n=3238) due to missing answers to some questions.

Use of at least one psychotropic medication was reported by 12.2% (n=396), 78.3% in women (n=310) and 21.7 in men (n=86). When stratified by sex, 14.0% (310/2221) of women and 8.5% (86/1017) of men used a psychotropic medication. A total of 518 psychotropic medications were identified (average 1.31 per user), with a higher prevalence of antidepressant use, and among antidepressants, selective serotonin reuptake inhibitors (SSRIs) were the most frequent (46.5%). According to generic name, the mostly used drugs were sertraline hydrochloride (15.4%), escitalopram oxalate (14.5%) and fluoxetine hydrochloride (9.7%) (Table 2).

**Table 2:** Distribution of psychotropic medications used by undergraduate students, according to generic name, GraduaUEL, 2019 (n=518).

Generic name	n	%
Sertraline hydrochloride	80	15.4
Escitalopram oxalate	75	14.5
Fluoxetine hydrochloride	50	9.7
Bupropion	29	5.6
Venlafaxine Hydrochloride	27	5.2
Desvenlafaxine Succinate	26	5.0
Zolpidem hemitartrate	19	3.7
Paroxetine hydrochloride	18	3.5
Trazodone hydrochloride	18	3.5
Clonazepam	16	3.1
Methylphenidate Hydrochloride	16	3.1
Duloxetine Hydrochloride	11	2.1
Quetiapine hemifumarate	11	2.1
Alprazolam	8	1.5
Amitriptyline Hydrochloride	8	1.5
Fluvoxamine	8	1.5
Risperidone	8	1.5
Melatonin	7	1.4
Passiflora incarnata	7	1.4
Citalopram bromide	6	1.2
Hydroxyzine hydrochloride	5	1.0
Vortioxetine	5	1.0

In the adjusted analysis, for the group of women, we found a statistically significant association between the higher frequency of use of psychotropic medications, access to health insurance (PR=1.49; 95%CI: 1.25-1.78), diagnosis of depression (PR=4.60; 95%CI: 3.55-5.95), diagnosis of anxiety (PR=4.58; 95%CI: 3.23-6.49) and use of illicit drugs in the last three months (PR=1.25; 95%CI: 1.02-1.53). In contrast, psychotropic medication use was less frequent in those women who consumed alcohol on a weekly basis (PR=0.74; 95%CI: 0.60-0.90) (Table 3).

**Table 3:** Association between independent variables and psychotropic drug use (crude and adjusted analysis) among university students, according to sex. GraduaUEL, 2019.

Independent variables		Use of psychotropic drugs			
		Women		Men	
		Crude analysis PR (95%CI)	Adjusted analysis ^a^ PR (95%CI)	Crude analysis PR (95%CI)	Adjusted analysis ^a^ PR (95%CI)
Age, β (95%CI)		0.033 (1.02-1.05)	0.008 (0.99-1.03)	-0.004 (0.96-1.03)	-0.035 (0.92-1.02)
Marital status					
	With partner	1	1	1	1
	Without partner	0.98 (0.79-1.20)	1.01 (0.84-1.21)	1.01 (0.67-1.52)	1.48 (0.99-2.24)
Self-reported skin color					
	Non-white	1	1	1	1
	White	1.16 (0.92-1.48)	1.14 (0.93-1.39)	1.02 (0.66-1.58)	1.06 (0.74-1.53)
Access to health insurance					
	No	1	1	1	1
	Yes	1.45 (1.17-1.78)	1.49 (1.25-1.78)	1.53 (1.02-2.29)	1.65 (1.15-2.36)
Study time					
	Morning/afternoon	1	1	1	1
	Night	1.11 (0.83-1.48)	0.97 (0.76- 1.23)	0.87 (0.47-1.62)	0.80 (0.45- 1.41)
	Full-time/LDE (long-distance education)	1.14 (0.89-1.47)	1.18 (0.95-1.47)	1.49 (0.88-2.54)	1.42 (0.84-2.41)
Year of study					
	1st year	1	1	1	1
	2nd or 3rd year	1.12 (0.84-1.50)	0.97 (0.76-1.23)	1.69 (0.95-3.02)	1.65 (0.94-2.89)
	4th, 5th or 6th year	1.39 (1.03-1.87)	1.18 (0.95-1.47)	1.53 (0.81-2.86)	1.60 (0.88-2.91)
Satisfaction with the course					
	Very satisfied/satisfied	1	1	1	1
	Neither satisfied nor dissatisfied	0.86 (0.66-1.12)	0.77 (0.61-1)	1.41 (0.85-2.35)	1.11 (0.71-1.73)
	Dissatisfied/very dissatisfied	1.62 (1.14-2.30)	0.81 (0.60-1.07)	2.23 (1.28-3.91)	1.28 (0.72-2.28)
Satisfaction with academic performance					
	Very satisfied/satisfied	1	1	1	1
	Neither satisfied nor dissatisfied	1.24 (0.96-1.60)	0.92 (0.74-1.14)	1.86 (1.13-3.06)	1.38 (0.87-2.19)
	Dissatisfied/very dissatisfied	1.62 (1.26-2.09)	1.05 (0.83-1.33)	2.15 (1.28-3.60)	1.77 (1.09-2.87)
Self-reported physical health status					
	Very good/good	1	1	1	1
	Regular	1.41 (1.09-1.84)	1.08 (0.85-1.36)	1.23 (0.76-1.99)	0.72 (0.44-1.17)
	Bad/very bad	2.02 (1.53-2.66)	0.95 (0.74-1.24)	2.55 (1.55-4.18)	1.14 (0.63-2.06)
Self-reported physical health status					
	Very good/good	1	1	1	1
	Regular	1.77 (1.27-2.46)	1.14 (0.83-1.55)	1.89 (1.07-3.32)	0.91 (0.49-1,69)
	Bad/very bad	3.72 (2.73-5.05)	1.28 (0.93-1.74)	4.33 (2.60-7.23)	1.12 (0.68-1.85)
Self-reported sleep quality					
	Very good/good	1	1	1	1
	Bad/very bad	1.53 (1.24-1.88)	0.94 (0.78-1.12)	1.39 (0.93-2.09)	0.98 (0.66-1.46)
Diagnosis of depression					
	No	1	1	1	1
	Yes	9.82 (8.01-11.91)	4.60 (3.55-5.95)	12.44 (8.69-17.79)	4.49 (2.81-7.16)
Anxiety diagnosis					
	No	1	1	1	1
	Yes	9.58 (7.17-12.81)	4.58 (3.23-6.49)	12.83 (7.88-20.87)	7.68 (4.25-13.89)
Alcohol consumption in the last three months					
	Never/once or twice/monthly	1	1	1	1
	Weekly	0.87 (0.69-1.11)	0.74 (0.60-0.90)	0.62 (0.39-1.01)	0.64 (0.41-1)
	Daily or almost daily	1.39 (0.76-2.52)	1.11 (0.64-1.90)	2.07 (1.02-4.20)	1.30 (0.57-2.99)
Illicit drug use in the last three months					
	Yes	1.40 (1.12-1.75)	1.25 (1.02-1.53)	1.16 (0.76-1.77)	1.01 (0.64-1.58)
	No/prefer not to answer	1	1	1	1

95% confidence interval: 95%CI; PR: prevalence ratio.

a Adjusted for all the variables in the table.

In the adjusted analysis for the group of men, we found that the use of psychotropic medications was more frequent among those with access to health insurance (PR=1.65; 95%CI: 1.15-2.36), dissatisfied or very dissatisfied with their academic performance (PR=1.77; 95%CI: 1.09-2.87), diagnosis of depression (PR=4.49; 95%CI: 2.81-7.16) and diagnosis of anxiety (PR=7.68; 95%CI: 4.25-13.89) (Table 3).

## DISCUSSION

This study identified a 12.0% frequency of psychotropic drug use among students at a public university in Paraná, Brazil. Regarding women, consumption was higher among those who reported having used illicit drugs in the last three months. In men, drug use was higher among students who reported being dissatisfied with their academic performance. As for the overall sample, the highest frequency of psychotropic drug use was found in those who had access to health insurance, diagnosis of depression and diagnosis of anxiety.

The frequency of psychotropic drug use found in this study is similar to that described in psychology students in Brazil (15.1%) ^20^^)^ and in students from different areas of a university in Portugal (12.7%) ^21^. A study conducted in students at the University of Lausanne, Switzerland, revealed that 12.1% of the participants reported having used psychotropic substances. In addition, women showed a higher propensity to use psychotropic drugs compared to men, which supports our findings ^22^. However, other works in Brazilian students from different health-related courses showed slightly higher prevalence rates (16.0%) ^23^^)^ (19.0%) ^24^, which is justified by the overload of academic and care activities ^2^^,^^3^^,^^9^.

Our study found that women were more likely to use psychotropic medications, a result consistent with previous research showing higher rates of antidepressant use among women compared with men. This gender difference in antidepressant use is a common finding in many studies, with possible implications for public health and clinical practice ^10^^,^^22^^,^^25^.

The use of psychotropic medications by women is a multifaceted phenomenon, influenced by a complex interaction of sociocultural, economic and health factors. In Uruguay, research has consistently shown that women are the main consumers of psychotropic medications, with a particular predilection for benzodiazepines and antidepressants. These medications are often used for extended periods of time, which may lead to a higher prevalence of psychotropic use among women. In addition, women are more likely to experience adverse side effects and to develop dependence on these medications, underscoring the need for a more nuanced understanding of the factors contributing to psychotropic use in this population ^26^.

A study conducted in Brazilian medical students revealed that 30.4% used psychotropic drugs, with anxiety and depression being the main reasons for prescription. A significant correlation was reported between academic progress and prevalence of psychotropic use, with a higher incidence of diagnoses of mental disorders among women. The research highlights the need for institutional interventions to promote mental health among these students, emphasizing the importance of adequate sleep and regular physical activity as protective factors ^10^.

The higher frequency of psychotropic medication use in women compared to men is justified, since depression, anxiety and stress conditions are more common in women ^10^^,^^19^^,^^27^^-^^29^. This research also highlights the higher frequency of medical diagnosis of depression and anxiety among female students, as well as the relationship of these diagnoses to psychotropic use. These medications are an important, although not the only, therapeutic strategy for the treatment of common mental disorders ^10^^,^^28^.

In addition, women recognize depressive symptoms better, report physical and psychological symptoms more easily, and seek help for health problems more frequently than men (19.30). According to the National Health Survey (PNS) (2019), women showed a higher rate (82.3%) of medical consultations compared to men (69.4%), making them more likely to use medication ^31^.

Antidepressants were the most frequently used class of drugs among students, with SSRIs being the most frequent subgroup, similar to what has been described by other studies ^3^. Regarding the mostly used psychotropic drugs, our results showed a higher frequency of sertraline hydrochloride, escitalopram oxalate and fluoxetine hydrochloride, aligning with the findings of other national and international works in university students ^3^^,^^21^^,^^23^^,^^32^^,^^33^. According to Martins de Oliveira *et al*. (2020) ^34^, 60% of university students in Brazil have experienced or are experiencing anxiety during their undergraduate studies. In addition, 32% reported suffering from insomnia, 30% have used or are using some type of psychiatric medication, 20% experience persistent sadness, 10% suffer from fear or panic, 6% have had suicidal ideation, and 4% have had suicidal thoughts. These health conditions reinforce the rationale for the use of antidepressants, which, in addition to treating depression, have also been widely used for anxiety and sleep disorders.

As for other factors associated with the use of psychotropic drugs, we highlight access to private health insurance. This relation is consistent with the Belo Horizonte Health Survey, conducted in the Metropolitan Region of Belo Horizonte, in the state of Minas Gerais, Brazil ^35^. Having private health insurance facilitates access to health services, particularly consultations with specialists, which contributes to greater use of medications, particularly psychotropic drugs, which cannot be purchased in pharmacies without a prescription ^21^^,^^35^.

Regarding dissatisfaction with academic performance, our study showed that students using psychotropic medications had a high prevalence of previous diagnosis of depression. Depression is considered a disabling condition that can interfere negatively in many spheres of life, including students’ academic performance ^3^. Berchtold *et al*. found that students who used psychotropic medications had lower academic performance, poorer health status, and lower life satisfaction compared with those who did not use them. In addition, the study suggests that academic pressure is related to the use of these substances, as drug users reported greater academic difficulties.

Regarding the relation between the use of illicit drugs and psychotropic medications among women, we consider that the overload of activities, more common among women ^36^, together with higher levels of stress, anxiety, depressive symptoms and the consequent use of medications for the central nervous system, favors the search for other forms of relief, such as the use of illicit drugs ^37^.

The relationship between illicit substance use and depression and anxiety is complex. Research has shown that illicit substance use can be a contributing factor to depression and anxiety, particularly when combined with other factors such as academic stress and performance pressure ^38^. In addition, the search for relief from symptoms of anxiety and depression may lead students to engage in substance use, including alcohol, tobacco, illicit drugs, and prescription medications ^39^.

Recent studies also found a higher prevalence of depressive and anxiety disorders among women, leading to the use of psychotropic medications, in addition to dependence on illicit drugs ^40^^-^^42^. Although drug use is more common among young men ^39^^,^^43^^,^^44^, it is possible that the use of these substances is less related to mental health conditions and, therefore, to psychotropic use in this population.

In contrast, weekly alcohol consumption was related to a lower use of psychotropic medications. Alcohol consumption is part of daily entertainment, facilitating social interaction among students. This interaction favors social support among them, thus reducing the chances of developing depressive and anxiety symptoms ^45^^,^^46^, and the consequent need for psychotropics. In contrast, a higher frequency and amount of alcohol consumption is associated with a higher risk of depression ^46^^,^^47^. This association was not identified in our study, but may be related to the fact that the nondrinker group included former drinkers who stopped drinking because of health problems or the need for psychotropic medications, which could confound the association. A study in Sweden found that those who consumed alcohol lightly and moderately were less likely to develop depression, whereas those who did not consume or consumed heavily were more likely to be depressed ^46^, which partially corroborates the findings of this research.

Our study has some limitations that should be highlighted. The data were collected online and, although widely disseminated, an electronic questionnaire does not allow a detailed explanation at the time of the survey. In this regard, the drug names may be subject to recall bias, despite the fact that this population is considered young and with a greater capacity to retain information. In addition, the response rate of this survey (25.8%) was lower than that reported by other studies with electronic questionnaires ^48^. It is also important to note that, although the researchers conducted a thorough review of the completed questionnaires, excluding duplicates, triplicates, and responses from graduate students, among others, the risk persists that individuals not affiliated with the undergraduate programs of the surveyed university may have completed the questionnaire. Finally, social desirability bias may occur in the research due to the tendency of participants to provide socially acceptable answers, which could distort their true opinions and behavior ^49^. As a strength, it should be noted that our study has a larger sample size than other surveys conducted among undergraduate students on medication use. In addition, it covers all areas and courses at UEL.

In conclusion, these results highlight the high frequency of psychotropic medication use among university students, especially among females, as well as among students with access to health insurance, with a diagnosis of depression and a diagnosis of anxiety, regardless of their sex. We also found that male students who reported dissatisfaction with their academic performance had a higher frequency of psychoactive medication use. Among the consumed medications, antidepressants, such as SSRIs, stood out, indicating the importance of investigating aspects related to mental health in this population. These findings demonstrate factors associated with the use of psychotropic medications in students, recognizing the university as an environment of increased susceptibility to mental health problems, which causes a greater need for central nervous system medications. We hope that this evidence can support actions at the local level, promoting not only campaigns for rational use of medications, but also measures to minimize and help students with the stress caused by academic life.
