# Supplementary material for: Factors associated with the use of psychotropic drugs by students at a brazilian public university
Source: Rev Peru Med Exp Salud Publica. 2024 Nov 26;41(4):365–74. doi: 10.17843/rpmesp.2024.414.13858 (PMC11797590; doi:10.17843/rpmesp.2024.414.13858)
Supplement: Translation

# Translation: Factores asociados al uso de psicofármacos por estudiantes de una universidad pública brasileña

**Authors:** Telma Regina Fares Gianjacomo, Camilo Molino Guidoni, Renne Rodrigues, Selma Maffei de Andrade, Jéssica Vertuan Rufino, Edmarlon Girotto

**Affiliations:** 1 Programa de Postgrado en Salud Pública, Universidad Estatal de Londrina (UEL), Londrina-PR, Brasil.; 2 Departamento de Ciencias Farmacéuticas, Universidad Estatal de Londrina (UEL), Londrina-PR, Brasil.; 3 Universidad Federal de la Frontera Sur (UFFS), Campus de Chapecó, Chapecó-SC, Brasil.

**Keywords:** Utilización de Medicamentos, Psicotrópicos, Salud del Estudiante, Universidad, Brasil

## Abstract

**Objetivo.:**

Analizar el consumo de medicamentos psicotrópicos y sus factores asociados en estudiantes universitarios, estratificado segun sexo.

**Materiales y métodos.:**

Se realizó un estudio transversal con estudiantes de pregrado de una universidad pública de Brasil. El instrumento fue un cuestionario autoadministrado en línea. La variable de resultado fue el uso de medicamentos psicotrópicos, con base en el sistema de clasificación Anatómico Terapéutico Químico (ATC) y las variables de exposición fueron socidemográficas, académicas y relacionadas con la salud. La asociación entre las variables de exposición y el resultado se verificaron mediante el cálculo de razones de prevalencia y sus intervalos de confianza al 95%.

**Resultados.:**

El 12,2% (n=396) de los 3238 participantes refirieron consumir psicofármacos, la mayoría fueron mujeres (78,3%). La prevalencia de consumo de psicotrópicos fue mayor entre los estudiantes con acceso a seguro de salud, diagnóstico de depresión y diagnóstico de ansiedad. También fue mayor en el grupo de mujeres que informaron consumir drogas ilícitas en los últimos tres meses y en el grupo de hombres que informaron estar insatisfechos o muy insatisfechos con su rendimiento académico. Se detectó una menor frecuencia de uso de psicofármacos entre las mujeres que consumen alcohol semanalmente.

**Conclusiones.:**

Los resultados indican una mayor frecuencia de consumo de medicamentos psicotrópicos en las mujeres, así como en los estudiantes con acceso al seguro de salud, diagnóstico de depresión y diagnóstico de ansiedad, independientemente del sexo.

## INTRODUCCIÓN

El ingreso a la educación superior es una transición que puede generar estrés, desequilibrio emocional, dificultades de adaptación y un gran impacto en la vida personal y académica ^(^^1^^).^ Factores inherentes al proceso académico son señalados como posibles causas de problemas de salud. Durante la universidad, algunos estudiantes se vuelven vulnerables a la aparición de trastornos afectivos, como depresión y ansiedad ^(^^2^. Una revisión sistemática con metaanálisis observó que el estrés, la baja frecuencia de actividades recreativas, la insatisfacción con el rendimiento académico y la falta de apoyo emocional en el entorno académico son factores de riesgo para problemas de salud mental en estudiantes universitarios de pregrado ^(^^3^.

Una investigación reciente sobre la salud mental de los estudiantes universitarios chilenos identificó una proporción significativa de estudiantes con problemas de salud mental, estimándose que entre el 20 y el 30% de este grupo se ve afectado por ansiedad, depresión y estrés. Además, se observa una notable disparidad de género, con una mayor prevalencia de síntomas depresivos en mujeres con 23,2%, en comparación con 13,4% en los hombres ^(^^4^. En este contexto, la salud mental de los estudiantes universitarios ha sido un tema de interés en la literatura ^(^^5^. Los estudios revelan una alta prevalencia combinada de depresión y ansiedad, entre otros problemas de salud mental, en esta población ^6^. En consecuencia, el uso de fármacos psicotrópicos entre estudiantes universitarios ha aumentado, especialmente antidepresivos, ansiolíticos y psicoestimulantes, siendo también más frecuente en mujeres ^(^^7^^-^^9^.

Un estudio realizado entre estudiantes universitarios descubrió una alta prevalencia de trastornos de salud mental, donde el 30,4% de los participantes informó el uso de medicamentos psicotrópicos, principalmente para el tratamiento de la ansiedad (30%) y la depresión (22,8%). El estudio reveló que el uso de medicamentos psicotrópicos se incrementa a medida que los estudiantes avanzan en sus programas académicos, particularmente en los últimos años, cuando se intensifican las presiones académicas. En cambio, la participación regular en actividades físicas y la adherencia a patrones de sueño adecuados fueron identificados como factores protectores contra los trastornos de salud mental, lo que subraya la necesidad de una mayor atención al bienestar mental de este grupo demográfico ^10^.

Para comprender mejor el uso de medicamentos psicotrópicos entre los estudiantes, es necesario considerar la presión del entorno académico, que contribuye al estrés y al deterioro de la salud mental. Además, es fundamental investigar cómo los factores individuales, como los antecedentes familiares y la resiliencia, interactúan con estos factores de estrés. También es pertinente analizar la relación entre el uso de psicotrópicos y los comportamientos de autocuidado. Un enfoque multidimensional que considere factores sociales y académicos puede ayudar a desarrollar intervenciones más efectivas para promover la salud mental entre los estudiantes ^11^^,^^12^.

Sin embargo, investigaciones sólidas sobre este tema son escasas. Por lo tanto, son necesarios estudios que caractericen el uso de medicamentos psicotrópicos entre estudiantes universitarios y que correlacionen variables que ayuden a identificar a los grupos más vulnerables a estos medicamentos. Además, algunos trabajos han mostrado que el consumo de fármacos psicoactivos es mayor en las mujeres, por lo que es importante evaluar la diferencia en los factores asociados entre estudiantes hombres y mujeres ^(^^13^^,^^14^. Por consiguiente, este estudio tiene como objetivo evaluar el uso de medicamentos psicotrópicos en estudiantes de una universidad pública en Paraná, Brasil, e identificar factores asociados sociodemográficos, académicos y de salud, estratificados segun sexo.

**MENSAJE CLAVE**

Motivación para realizar el estudio. Los estudiantes universitarios están expuestos a numerosos eventos estresantes, que los predisponen a problemas como la depresión y la ansiedad, lo que lleva a un aumento en el consumo de medicamentos psicotrópicos.

Principales hallazgos. El uso de medicamentos psicotrópicos fue reportado por el 12,0% de los estudiantes, siendo mayor entre aquellos con acceso a seguro de salud y diagnosticados con depresión y ansiedad, así como entre los que informaron utilizar drogas ilícitas y que estaban insatisfechos con su rendimiento académico. Entre las mujeres que consumían bebidas alcohólicas hubo menor consumo de psicofármacos.

Implicancias. La evidencia de este estudio puede respaldar acciones para promover no solo campañas de uso racional de medicamentos, sino también medidas para minimizar y ayudar a los estudiantes con el estrés de la vida académica.

## MATERIALES Y MÉTODOS

### Diseño del estudio, ubicación y población

Este es un estudio transversal, guiado por las pautas de Strengthening the Reporting of Observational Studies in Epidemiology (STROBE) ^(^^15^, que analiza los datos del proyecto «GraduaUEL - Análisis de la Salud y Hábitos de Vida de Estudiantes de Pregrado en la UEL». El objetivo general de GraduaUEL fue analizar aspectos relacionados con la salud, la exposición a la violencia y los hábitos de vida de los estudiantes universitarios. La población del estudio está compuesta por estudiantes de la Universidad Estatal de Londrina (UEL), en Paraná, Brasil, mayores de 18 años, regularmente matriculados en el primer semestre del 2019 en uno de los 51 cursos de pregrado. En el momento de la encuesta, 12 536 estudiantes eran elegibles para participar ^(^^16^.

### Preprueba y estudio piloto

El cuestionario preparado para esta investigación se puso a disposición en la plataforma Google Forms® para que los estudiantes lo completaran. El cuestionario se dividió en varias secciones temáticas, que abarcó la caracterización general y académica, hábitos de vida y calidad del sueño, uso de medicamentos, experiencias de violencia, apoyo social y resiliencia, salud mental y satisfacción corporal. Para garantizar su validez para la aplicación dentro de la población de estudio, el instrumento fue evaluado por expertos en investigación epidemiológica. Se realizó una preprueba con 25 estudiantes de pregrado del área de salud en una institución privada de la ciudad de Londrina, Brasil, para evaluar la claridad de las preguntas. Además, se llevó a cabo un estudio piloto con 25 estudiantes de un instituto federal de educación superior en la región de Londrina, Paraná. Esta etapa se realizó para verificar las condiciones logísticas para la recolección de datos, tales como el tiempo de respuesta y el rendimiento de la plataforma durante el acceso simultáneo.

### Recolección de datos

Los datos fueron recolectados entre abril y junio del 2019. Durante este período, los investigadores promovieron el estudio en la UEL, en todas las clases de pregrado, proporcionando el enlace para acceder al cuestionario. También se realizó una amplia divulgación en redes sociales, prensa local y correos electrónicos masivos enviados a la comunidad universitaria. El cuestionario se respondió de forma anónima, y la opción de completar el número de matrícula era opcional para una futura retroalimentación individual. Se instruyó a los participantes a responder el cuestionario solo una vez. Además, antes de que los estudiantes consintieran participar en el estudio, se les preguntaba, en la página inicial del cuestionario electrónico, si estaban matriculados en un programa de pregrado. Si la respuesta era «no», no se mostraban las opciones del cuestionario. En los casos donde se identificaron respuestas duplicadas o triplicadas, solo se consideró la primera respuesta. Cuando el número de matrícula indicaba que los estudiantes eran de posgrado, estos fueron excluídos del estudio.

### Variable de resultado

El uso de medicamentos psicotrópicos fue la variable de resultado que fue evaluada mediante la siguiente pregunta: «¿Toma algún medicamento de uso contínuo?» Si la respuesta era afirmativa, se solicitaba el nombre del medicamento, la persona responsable de la prescripción y el período de uso. Tras la recolección de datos, los nombres de los medicamentos fueron estandarizados a su nombre genérico y categorizados de acuerdo con el sistema de clasificación Anatomical Therapeutic Chemical (ATC) de la Organización Mundial de la Salud, considerando los siguientes subgrupos como psicotrópicos: N05A (antipsicóticos), N05B (ansiolíticos), N05C (hipnóticos y sedantes), N06A (antidepresivos), N06B (psicoestimulantes y agentes para TDAH y nootrópicos) y N06D (medicamentos antidemencia) ^(^^17^. Los medicamentos utilizados fueron verificados en duplicado por diferentes investigadores y luego analizados con el software Epi Info, versión 3.5.1. En caso de discrepancias, un tercer investigador realizó las correcciones necesarias.

### Variables de exposición

Se utilizaron variables sociodemográficas, académicas, de estilo de vida y relacionadas con la salud como variables de exposición. Las variables sociodemográficas evaluadas fueron: edad (años), estado civil (con pareja-casado o en unión estable; sin pareja-soltero, divorciado o viudo), color de piel autodeclarado (blanco; no blanco-amarillo, multirracial, negro o indígena) y acceso a seguro de salud (sí; no). Las variables académicas analizadas fueron: turno de estudio (mañana/tarde; noche; tiempo completo/EAD-educación a distancia), año de estudio (1º año; 2º o 3º año; 4º, 5º o 6º año) y satisfacción con el curso, y el rendimiento académico (muy satisfecho/satisfecho; ni satisfecho ni insatisfecho; insatisfecho/muy insatisfecho). Finalmente, las variables relacionadas con el estilo de vida y los hábitos de salud fueron: estado de salud física y mental autodeclarado (muy bueno/bueno; regular; malo/muy malo), calidad del sueño autodeclarada (muy buena/buena; mala/muy mala), diagnóstico médico autodeclarado de depresión y ansiedad (sí; no), consumo de alcohol (nunca/una o dos veces/ocasionalmente; semanalmente; diariamente o casi diariamente) y uso de sustancias ilícitas en los últimos tres meses (sí; no o prefiero no responder). La selección de las variables para ser evaluados como asociados con el uso de medicamentos psicotrópicos se basó en la literatura, especialmente con estudios relacionados con trastornos mentales ^6^^,^^18^, que sirven como indicadores para el uso de sustancias psicotrópicas. La excepción fueron las variables académicas, que se seleccionaron con base en las variables disponibles en la investigación. 

### Análisis estadístico

Los datos fueron analizados utilizando el paquete estadístico Statistical Package for the Social Sciences (SPSS® versión 19.0). Para la caracterización de la población de estudio, se realizó un análisis descriptivo, presentando las frecuencias de las variables cuantitativas categorizadas por sexo (hombre y mujer) ^19^. También se calcularon la media de la edad y la desviación estándar para cada sexo de forma independiente. Se utilizó la regresión de Poisson con varianza robusta para analizar la asociación entre el resultado y las variables de exposición, y obtener razones de prevalencia (RP) como medida de asociación e intervalos de confianza del 95% (IC95%). Se realizaron análisis crudos (o bivariado) y ajustados, incluyendo todas las variables independientes analizadas. Todas las variables independientes fueron incluidas en el modelo ajustado, considerando que, excepto por las variables académicas, todas están respaldadas por la literatura como asociadas a trastornos mentales o al uso de medicamentos psicotrópicos. El nivel de significancia adoptado fue del 5% (valor de p<0,05).

### Consideraciones éticas

Todos los sujetos de investigación aceptaron participar en el estudio, ya que solo era posible responder el cuestionario si aceptaban el formulario de consentimiento informado. Este estudio fue aprobado por el Comité de Ética en Investigación con Seres Humanos (CEP) de la UEL (CAAE nº 04456818.0.0000.5231).

## RESULTADOS

Se incluyeron 3238 estudiantes según la selección mostrada en la figura 1. La mayoría de los estudiantes participantes en el estudio se autodeclararon blancos y sin pareja. Los hombres mostraron porcentajes más altos de buen estado de salud físico, calidad del sueño y salud mental autodeclarados en comparación con las mujeres (Tabla 1).

**Figura 1:** Diagrama de flujo de la muestra del estudio GraduaUEL, 2019

**Tabla 1:** Descripción de las variables académicas, hábitos de vida y condiciones de salud de los estudiantes universitarios, según sexo. GraduaUEL, 2019.

Variables independientes ^a^		Mujeres		Hombres	
		Total n=2221	Uso de medicamentos psicotrópicos n=310 (78,3%)	Total n=1017	Uso de medicamentos psicotrópicos n=86 (21,7%)
		n (%)	n (%)	n (%)	n (%)
Edad (media ± desviación estándar)		21,76 ± 4,37	22,55 ± 4,90	22,28 ± 4,67	22,17 ± 3,74
Estado civil (n=3238)					
	Con pareja	1107 (49,8)	158 (14,2)	413 (40,6)	51 (8,4)
	Sin pareja	1114 (50,2)	152 (13,7)	604 (59,4)	35 (8,5)
Color de piel autodeclarado (n=3236)					
	Blanco	1565 (70,5)	228 (14,6)	693 (68,1)	59 (8,5)
	No blanco	654 (29,5)	82 (12,5)	324 (31,9)	27 (8,3)
Acceso a seguro de salud (n=3235)					
	No	1194 (53,8)	138 (11,6)	591 (58,2)	41 (6,9)
	Sí	1025 (46,2)	171 (16,7)	425 (41,8)	45 (10,6)
Período de estudio (n=3236)					
	Mañana/tarde	642 (28,9)	82 (12,8)	235 (23,1)	17 (7,2)
	Noche	582 (26,2)	83 (14,3)	337 (33,1)	21 (6,2)
	Tiempo completo/EAD (educación a distancia)	995 (44,9)	145 (14,6)	445 (43,8)	48 (10,8)
Año de estudio (n=3226)					
	1º año	477 (21,5)	56 (11,7)	243 (24,0)	14 (5,8)
	2º o 3º año	1004 (45,4)	133 (12,2)	473 (46,8)	46 (9,7)
	4º, 5º o 6º año	733 (33,1)	129 (16,4)	296 (29,2)	26 (8,8)
Satisfacción con el curso (n=3235)					
	Muy satisfecho/satisfecho	1598 (72,0)	222 (13,9)	760 (74,8)	55 (7,2)
	Ni satisfecho ni insatisfecho	496 (22,4)	60 (12,1)	175 (17,2)	18 (10,3)
	Insatisfecho/muy insatisfecho	125 (5,6)	28 (22,4)	81 (8,0)	13 (16,0)
Satisfacción con el rendimiento académico (n=3236)					
	Muy satisfecho/satisfecho	916 (41,2)	104 (11,4)	456 (44,9)	25 (5,5)
	Ni satisfecho ni insatisfecho	756 (34,1)	106 (14,0)	324 (31,9)	33 (10,2)
	Insatisfecho/muy insatisfecho	548 (24,7)	110 (18,2)	236 (23,2)	28 (11,9)
Estado de salud física autodeclarado (n=3238)					
	Muy bueno/bueno	785 (35,3)	78 (9,9)	486 (47,8)	31 (6,4)
	Regular	952 (42,9)	134 (14,1)	379 (37,3)	30 (7,9)
	Malo/muy malo	484 (21,8)	98 (20,2)	152 (14,9)	25 (16,4)
Estado de salud mental autodeclarado (n=3238)					
	Muy bueno/bueno	715 (32,2)	47 (6,6)	475 (46,7)	20 (4,2)
	Regular	848 (38,2)	101 (11,9)	325 (32,0)	26 (8,0)
	Malo/muy malo	658 (29,6)	162 (24,9)	217 (21,3)	40 (18,4)
Calidad del sueño autodeclarada (n=3238)					
	Muy buena/buena	1171 (52,7)	131 (11,2)	581 (57,1)	42 (7,2)
	Mala/muy mala	1050 (47,3)	179 (17,0)	436 (42,9)	44 (10,1)
Diagnóstico médico de depresión (n=3236)					
	No	1919 (86,5)	123 (6,4)	935 (91,9)	41 (4,4)
	Sí	300 (13,5)	187 (62,3)	82 (8,1)	45 (54,9)
Diagnóstico médico de ansiedad (n=3236)					
	No	1444 (65,0)	50 (3,5)	799 (78,6)	19 (2,4)
	Sí	777 (35,0)	260 (33,5)	218 (21,4)	67 (30,7)
Consumo de alcohol en los últimos tres meses (n=3238)					
	Nunca/una o dos veces/mensualmente	1527 (68,8)	219 (14,3)	619 (60,9)	58 (9,4)
	Semanalmente	647 (29,1)	81 (12,5)	362 (35,6)	21 (5,8)
	Diariamente o casi diariamente	47 (2,1)	10 (21,3)	36 (3,5)	7 (19,4)
Uso de drogas ilícitas en los últimos tres meses (n=3238)					
	No/prefiero no responder	1698 (76,5)	217 (12,8)	695 (68,3)	56 (8,1)
	Sí	523 (23,5)	93 (17,8)	322 (31,7)	30 (9,3)

a El número total de encuestados para algunas variables fue diferente del total de participantes (n=3238) debido a respuestas faltantes en algunas preguntas.

El uso de al menos un medicamento psicotrópico fue reportado por el 12,2% (n=396), 78,3% en mujeres (n=310) y 21,7 en hombres (n=86). Cuando se estratifico por sexo, 14,0% (310/2221) de las mujeres y 8,5% (86/1017) de los hombres usaron un medicamento psicotrópico. Se identificaron un total de 518 medicamentos psicotrópicos (promedio de 1,31 por usuario), con una mayor prevalencia en el uso de antidepresivos, y entre los antidepresivos, los inhibidores selectivos de la recaptación de serotonina (ISRS) fueron los más frecuentes (46,5%). Según el nombre genérico, los principales medicamentos utilizados fueron clorhidrato de sertralina (15,4%), oxalato de escitalopram (14,5%) y clorhidrato de fluoxetina (9,7%) (Tabla 2).

**Tabla 2:** Distribución de medicamentos psicotrópicos utilizados por estudiantes de pregrado, según el nombre genérico, GraduaUEL, 2019 (n=518).

Nombre genérico	n	%
Clorhidrato de sertralina	80	15,4
Oxalato de escitalopram	75	14,5
Clorhidrato de fluoxetina	50	9,7
Bupropión	29	5,6
Clorhidrato de venlafaxina	27	5,2
Succinado de desvenlafaxina	26	5,0
Hemitartrato de zolpidem	19	3,7
Clorhidrato de paroxetina	18	3,5
Clorhidrato de trazodona	18	3,5
Clonazepam	16	3,1
Clorhidrato de metilfenidato	16	3,1
Clorhidrato de duloxetina	11	2,1
Hemifumarato de quetiapina	11	2,1
Alprazolam	8	1,5
Clorhidrato de amitriptilina	8	1,5
Fluvoxamina	8	1,5
Risperidona	8	1,5
Melatonina	7	1,4
Passiflora incarnata	7	1,4
Bromuro de citalopram	6	1,2
Clorhidrato de hidroxicina	5	1,0
Vortioxetina	5	1,0

En el análisis ajustado, para el grupo de mujeres se observó una asociación estadísticamente significativa entre la mayor frecuencia de uso de medicamentos psicotrópicos, acceso a seguro de salud (RP=1,49; IC95%: 1,25-1,78), diagnóstico de depresión (RP=4,60; IC95%: 3,55-5,95), diagnóstico de ansiedad (RP=4,58; IC95%: 3,23-6,49) y uso de drogas ilícitas en los últimos tres meses (RP=1,25; IC95%: 1,02-1,53). En contraste, el uso de medicamentos psicotrópicos fue menos frecuente en aquellas mujeres que consumen alcohol semanalmente (RP=0,74; IC95%: 0,60-0,90) (Tabla 3).

**Tabla 3:** Asociación entre variables independientes y consumo de psicofármacos (análisis crudo y ajustado) entre estudiantes universitarios, según sexo. Gradua UEL, 2019.

Variables independientes		Uso de medicamentos psicotrópicos			
		Mujeres		Hombres	
		Análisis crudo RP (IC95%)	Análisis ajustado ^a^ RP (IC95%)	Análisis crudo RP (IC95%)	Análisis ajustado ^a^ RP (IC95%)
Edad, β (IC95%)		0,033 (1,02-1,05)	0,008 (0,99-1,03)	-0,004 (0,96-1,03)	-0,035 (0,92-1,02)
Estado civil					
	Con pareja	1	1	1	1
	Sin pareja	0,98 (0,79-1,20)	1,01 (0,84-1,21)	1,01 (0,67-1,52)	1,48 (0,99-2,24)
Color de piel autodeclarado					
	No blanco	1	1	1	1
	Blanco	1,16 (0,92-1,48)	1,14 (0,93-1,39)	1,02 (0,66-1,58)	1,06 (0,74-1,53)
Acceso a seguro de salud					
	No	1	1	1	1
	Sí	1,45 (1,17-1,78)	1,49 (1,25-1,78)	1,53 (1,02-2,29)	1,65 (1,15-2,36)
Período de estudio					
	Mañana/tarde	1	1	1	1
	Noche	1,11 (0,83-1,48)	0,97 (0,76- 1,23)	0,87 (0,47-1,62)	0,80 (0,45- 1,41)
	Tiempo completo/EAD (educación a distancia)	1,14 (0,89-1,47)	1,18 (0,95-1,47)	1,49 (0,88-2,54)	1,42 (0,84-2,41)
Año de estudio					
	1º año	1	1	1	1
	2º o 3º año	1,12 (0,84-1,50)	0,97 (0,76-1,23)	1,69 (0,95-3,02)	1,65 (0,94-2,89)
	4º, 5º o 6º año	1,39 (1,03-1,87)	1,18 (0,95-1,47)	1,53 (0,81-2,86)	1,60 (0,88-2,91)
Satisfacción con el curso					
	Muy satisfecho/satisfecho	1	1	1	1
	Ni satisfecho ni insatisfecho	0,86 (0,66-1,12)	0,77 (0,61-1)	1,41 (0,85-2,35)	1,11 (0,71-1,73)
	Insatisfecho/muy insatisfecho	1,62 (1,14-2,30)	0,81 (0,60-1,07)	2,23 (1,28-3,91)	1,28 (0,72-2,28)
Satisfacción con el rendimiento academico					
	Muy satisfecho/satisfecho	1	1	1	1
	Ni satisfecho ni insatisfecho	1,24 (0,96-1,60)	0,92 (0,74-1,14)	1,86 (1,13-3,06)	1,38 (0,87-2,19)
	Insatisfecho/muy insatisfecho	1,62 (1,26-2,09)	1,05 (0,83-1,33)	2,15 (1,28-3,60)	1,77 (1,09-2,87)
Estado de salud física autodeclarado					
	Muy bueno/bueno	1	1	1	1
	Regular	1,41 (1,09-1,84)	1,08 (0,85-1,36)	1,23 (0,76-1,99)	0,72 (0,44-1,17)
	Malo/muy malo	2,02 (1,53-2,66)	0,95 (0,74-1,24)	2,55 (1,55-4,18)	1,14 (0,63-2,06)
Estado de salud física autodeclarado					
	Muy bueno/bueno	1	1	1	1
	Regular	1,77 (1,27-2,46)	1,14 (0,83-1,55)	1,89 (1,07-3,32)	0,91 (0,49-1,69)
	Malo/muy malo	3,72 (2,73-5,05)	1,28 (0,93-1,74)	4,33 (2,60-7,23)	1,12 (0,68-1,85)
Calidad del sueño autodeclarada					
	Muy buena/buena	1	1	1	1
	Mala/muy mala	1,53 (1,24-1,88)	0,94 (0,78-1,12)	1,39 (0,93-2,09)	0,98 (0,66-1,46)
Diagnóstico de depresión					
	No	1	1	1	1
	Sí	9,82 (8,01-11,91)	4,60 (3,55-5,95)	12,44 (8,69-17,79)	4,49 (2,81-7,16)
Diagnóstico de ansiedad					
	No	1	1	1	1
	Sí	9,58 (7,17-12,81)	4,58 (3,23-6,49)	12,83 (7,88-20,87)	7,68 (4,25-13,89)
Consumo de alcohol en los últimos tres meses					
	Nunca/una o dos veces/mensualmente	1	1	1	1
	Semanalmente	0,87 (0,69-1,11)	0,74 (0,60-0,90)	0,62 (0,39-1,01)	0,64 (0,41-1)
	Diariamente o casi diariamente	1,39 (0,76-2,52)	1,11 (0,64-1,90)	2,07 (1,02-4,20)	1,30 (0,57-2,99)
Uso de drogas ilícitas en los últimos tres meses					
	Sí	1,40 (1,12-1,75)	1,25 (1,02-1,53)	1,16 (0,76-1,77)	1,01 (0,64-1,58)
	No/prefiero no responder	1	1	1	1

RP: razón de prevalencia; IC95%: intervalo de confianza al 95%.

a Ajustado por todas las variables de la tabla.

En el análisis ajustado para el grupo de hombres, se observó que el uso de medicamentos psicotrópicos fue más frecuente entre aquellos con acceso a seguro de salud (RP=1,65; IC95%: 1,15-2,36), insatisfechos o muy insatisfechos con el rendimiento académico (RP=1,77; IC95%: 1,09-2,87), diagnóstico de depresión (RP=4,49; IC95%: 2,81-7,16) y diagnóstico de ansiedad (RP=7,68; IC95%: 4,25-13,89) (Tabla 3).

## DISCUSIÓN

Este estudio identificó una frecuencia de 12,0% de uso de medicamentos psicotrópicos entre estudiantes de una universidad pública en Paraná, Brasil. En las mujeres, el consumo fue mayor entre aquellas que informaron haber usado drogas ilícitas en los últimos tres meses. En los hombres, el consumo fue más alto entre los estudiantes que se declararon insatisfechos con su rendimiento académico. En cuanto a la muestra general, la mayor frecuencia de uso de medicamentos psicotrópicos se observó en aquellos que tenían acceso a seguro de salud, diagnóstico de depresión y diagnóstico de ansiedad.

La frecuencia de uso de medicamentos psicotrópicos hallada en este estudio es similar a lo descrito en estudiantes de psicología en Brasil (15,1%) ^20^ y en estudiantes de diferentes áreas de una universidad en Portugal (12,7%) ^(^^21^. Un trabajo realizado en estudiantes de la Universidad de Lausana, Suiza, reveló que el 12,1% de los participantes informaron haber usado sustancias psicotrópicas. Además, las mujeres mostraron una mayor propensión al uso de medicamentos psicotrópicos en comparación con los hombres, lo que respalda nuestros hallazgos ^(^^22^. Sin embargo, otros trabajos en estudiantes brasileños de diferentes cursos de salud mostraron tasas de prevalencia ligeramente más altas (16,0%) ^23^ (19,0%) ^(^^24^, lo cual se justifica por la sobrecarga de actividades académicas y asistenciales ^2^^,^^3^^,^^9^.

Nuestro estudio encontró que las mujeres tenían más probabilidades de usar medicamentos psicotrópicos, un resultado consistente con investigaciones previas que muestran tasas más altas de uso de antidepresivos entre mujeres en comparación con los hombres. Esta diferencia de género en el uso de antidepresivos es un hallazgo común en muchos estudios, con posibles implicaciones para la salud pública y la práctica clínica ^(^^10^^,^^22^^,^^25^.

El uso de medicamentos psicotrópicos por mujeres es un fenómeno multifacético, influido por una compleja interacción de factores socioculturales, económicos y de salud. En Uruguay, la investigación ha demostrado consistentemente que las mujeres son las principales consumidoras de medicamentos psicotrópicos, con una predilección particular por las benzodiacepinas y antidepresivos. Estos medicamentos suelen usarse por períodos prolongados, lo que puede llevar a una mayor prevalencia de uso de psicotrópicos entre las mujeres. Además, las mujeres son más propensas a experimentar efectos secundarios adversos y a desarrollar dependencia a estos medicamentos, lo que subraya la necesidad de una comprensión más matizada de los factores que contribuyen al uso de psicotrópicos en esta población ^(^^26^.

Un estudio realizado entre estudiantes de medicina brasileños reveló que el 30,4% usaba medicamentos psicotrópicos, siendo la ansiedad y la depresión las principales razones para la prescripción. Se observó una correlación significativa entre el progreso en el curso y la prevalencia de uso de psicotrópicos, con una mayor incidencia de diagnósticos de trastornos mentales entre las mujeres. La investigación destaca la necesidad de intervenciones institucionales para promover la salud mental entre estos estudiantes, enfatizando la importancia de un sueño adecuado y de la actividad física regular como factores protectores ^(^^10^.

 La mayor frecuencia de uso de medicamentos psicotrópicos en mujeres en comparación con los hombres se justifica, ya que las condiciones de depresión, ansiedad y estrés son más comunes en las mujeres ^(^^10^^,^^19^^,^^27^^-^^29^. Esta investigación también destaca la mayor frecuencia de diagnóstico médico de depresión y ansiedad entre las estudiantes, así como la relación de estos diagnósticos con el uso de psicotrópicos. Estos medicamentos son una estrategia terapéutica importante, aunque no la única, para el tratamiento de trastornos mentales comunes ^(^^10^^,^^28^.

Además, las mujeres reconocen mejor los síntomas depresivos, informan síntomas físicos y psicológicos con mayor facilidad, y buscan ayuda para problemas de salud más frecuentemente que los hombres ^(^^19^^,^^30^. Según la Encuesta Nacional de Salud (PNS) (2019), las mujeres mostraron una mayor proporción (82,3%) de consultas médicas en comparación con los hombres (69,4%), lo que las hace más propensas al uso de medicamentos ^31^.

Los antidepresivos fueron la clase de medicamentos más utilizados entre los estudiantes, siendo los ISRS el subgrupo más frecuente, de forma similar a lo descrito en otros estudios ^3^. En cuanto a los principales psicofármacos utilizados, los resultados mostraron una mayor frecuencia de clorhidrato de sertralina, oxalato de escitalopram y clorhidrato de fluoxetina, alineándose con los hallazgos de otros trabajos nacionales e internacionales en estudiantes universitarios ^3^^,^^21^^,^^23^^,^^32^^,^^33^. Según Martins de Oliveira *et al*. (2020) ^(^^34^, el 60% de los estudiantes universitarios en Brasil ha experimentado o está experimentando ansiedad durante sus estudios de pregrado. Además, el 32% informó sufrir de insomnio, el 30% ha usado o está usando algún tipo de medicación psiquiátrica, el 20% experimenta tristeza persistente, el 10% sufre de miedo o pánico, el 6% ha tenido ideación suicida y el 4% ha tenido pensamientos suicidas. Estas condiciones de salud refuerzan la razón para el uso de antidepresivos, los cuales, además de tratar la depresión, también se han utilizado ampliamente para los trastornos de ansiedad y del sueño.

En cuanto a otros factores asociados al uso de medicamentos psicotrópicos, destacamos el acceso a seguro de salud privado. Esta relación concuerda con la Encuesta de Salud de Belo Horizonte, realizada en la Región Metropolitana de Belo Horizonte, en el estado de Minas Gerais de Brasil ^35^. La tenencia de un seguro de salud privado facilita el acceso a servicios de salud, especialmente consultas con especialistas, lo que contribuye a un mayor uso de medicamentos, en particular psicotrópicos, que no pueden adquirirse en farmacias sin receta médica ^21^^,^^35^.

En lo que respecta a la insatisfacción con el rendimiento académico, el presente estudio mostró que los estudiantes que usaban medicamentos psicotrópicos presentaban una alta prevalencia de diagnóstico previo de depresión. La depresión es considerada una condición incapacitante que puede interferir negativamente en diversas esferas de la vida, incluido el rendimiento académico de los estudiantes ^(^^3^. Berchtold *et al*. encontraron que los estudiantes que usaban medicamentos psicotrópicos tenian un rendimiento académico más bajo, peor estado de salud y menor satisfacción con la vida en comparación con aquellos que no los usaban. Además, el estudio sugiere que la presión académica está relacionada con el uso de estas sustancias, ya que los usuarios de medicamentos informaron mayores dificultades académicas.

En cuanto a la relación entre el uso de drogas ilícitas y medicamentos psicotrópicos entre mujeres, consideramos que la sobrecarga de actividades, más común entre las mujeres ^(^^36^, junto con niveles más altos de estrés, ansiedad, síntomas depresivos y el consecuente uso de medicamentos para el sistema nervioso central, favorece la búsqueda de otras formas de alivio, como el uso de drogas ilícitas ^37^.

La relación entre el uso de sustancias ilícitas y la depresión y ansiedad es compleja. La investigación ha demostrado que el uso de sustancias ilícitas puede ser un factor que contribuye a la depresión y ansiedad, particularmente cuando se combina con otros factores como el estrés académico y la presión por el rendimento ^38^. Además, la búsqueda de alivio de los síntomas de ansiedad y depresión puede llevar a los estudiantes a involucrarse en el consumo de sustancias, incluyendo alcohol, tabaco, drogas ilícitas y medicamentos prescritos ^39^.

Estudios recientes también encontraron una mayor prevalencia de trastornos depresivos y de ansiedad entre mujeres, lo que lleva al uso de medicamentos psicotrópicos, además de la dependencia de drogas ilícitas ^40^^-^^42^. Aunque el consumo de drogas es más común entre los hombres jóvenes ^39^^,^^43^^,^^44^, es posible que el uso de estas sustancias esté menos relacionado con condiciones de salud mental y, por lo tanto, con el uso de psicotrópicos en esta población.

Por el contrario, el consumo semanal de alcohol se relacionó con un menor uso de medicamentos psicotrópicos. El consumo de alcohol forma parte del entretenimiento cotidiano, facilitando la interacción social de los estudiantes. Esta interacción favorece el apoyo social entre ellos, reduciendo así las posibilidades de desarrollar síntomas depresivos y de ansiedad ^45^^,^^46^, y la consecuente necesidad de psicotrópicos. En contraste, una mayor frecuencia y cantidad de consumo de alcohol se asocia con un mayor riesgo de depresión ^(^^46^^,^^47^. Esta asociación no fue identificada en el presente estudio, pero puede estar relacionada con el hecho de que el grupo de no bebedores incluía exbebedores que dejaron de consumir debido a problemas de salud o a la necesidad de medicamentos psicotrópicos, lo que podría confundir la asociación. Un estudio en Suecia encontró que aquellos que consumían alcohol de forma ligera y moderada tenían menos probabilidades de desarrollar depresión, mientras que aquellos que no consumían o consumían en exceso presentaban una mayor probabilidad de depresión ^(^^46^, lo que corrobora parcialmente los hallazgos de esta investigación.

Algunas limitaciones del presente estudio deben ser destacadas. Los datos fueron recolectados en línea y, aunque ampliamente divulgados, un cuestionario electrónico no permite una explicación detallada en el momento de la encuesta. En este sentido, el registro del nombre del medicamento puede estar sujeto a sesgo de memoria, a pesar de que esta población es considerada joven y con mayor capacidad de retener información. Además, la tasa de respuesta de esta encuesta (25,8%) fue menor que la observada en otros estudios con cuestionarios electrónicos ^48^. Asimismo, es importante destacar que, aunque los investigadores realizaron una revisión exhaustiva de los cuestionarios completados, excluyendo duplicados, triplicados y respuestas de estudiantes de posgrado, entre otros, persiste el riesgo de que individuos no afiliados a los programas de pregrado de la universidad encuestada hayan completado el cuestionario. Por último, puede ocurrir sesgo de deseabilidad social en la investigación debido a la tendencia de los participantes a proporcionar respuestas socialmente aceptables, lo que podría distorsionar sus verdaderas opiniones y comportamiento ^49^. Como fortaleza se debe destacar que el presente estudio cuenta con un tamaño de muestra mayor que otras encuestas realizadas entre estudiantes de pregrado sobre el uso de medicamentos. Además, abarca todas las áreas y cursos de la UEL.

En conclusión, estos resultados destacan una alta frecuencia del uso de medicamentos psicotrópicos entre los estudiantes universitarios, especialmente entre las mujeres, así como entre los estudiantes con acceso a seguro de salud, con diagnóstico de depresión y diagnóstico de ansiedad, independientemente de su sexo. También se encontró que los estudiantes varones que reportaron insatisfacción con su rendimiento académico tenían una mayor frecuencia de uso de medicamentos psicoactivos. Entre los medicamentos consumidos, los antidepresivos, como los ISRS, se destacaron, lo que indica la importancia de investigar aspectos relacionados con la salud mental en esta población. Estos hallazgos demuestran los factores asociados con el uso de medicamentos psicotrópicos en los estudiantes, reconociendo a la universidad como un entorno de mayor susceptibilidad a problemas de salud mental, lo que genera una mayor necesidad de medicamentos para el sistema nervioso central. Esperamos que esta evidencia pueda respaldar acciones a nivel local, promoviendo no solo campañas de uso racional de medicamentos, sino también medidas para minimizar y ayudar a los estudiantes con el estrés causado por la vida académica.
